# Supplementary material for: TcpC Inhibits M1 but Promotes M2 Macrophage Polarization via Regulation of the MAPK/NF-κB and Akt/STAT6 Pathways in Urinary Tract Infection
Source: Cells. 2022 Aug 28;11(17):2674. doi: 10.3390/cells11172674 (PMC9454685; doi:10.3390/cells11172674)
Supplement: Supplementary file 1 [file cells-11-02674-s001.zip › cells-1866126-Supplementary Material.pdf]

TcpC inhibits M1 but promotes M2 macrophage polarization *via* regulation of the MAPK/NF-κB and Akt/STAT6 pathways in urinary tract infection

Jia-qi Fang[1,2,#], Qian Ou[3,#], Bo-heng Wu[2,#], Si-si Li[1,2], Mian Wu[4], Jia-ling Qiu[1,2], Nuo Cen[1,2], Kai-xin Hu[1,2], Yang-fei Che[1,2], Yuan Ma[2], Jian-ping Pan[1,2]

Supplementary information

1. Materials and methods

1.1. Preparation of K-macrophages

K-macrophages from mouse models were prepared as previously described [1]. K-macrophages were prepared as follows: 1) Outer capsule of kidneys from control or model mice was removed. Kidneys were perfused with PBS to remove the blood in the glomeruli, and then kidneys were cut into small pieces and put into a 1.5 ml tube. 2) 1 ml of the collagenase solution was added and then incubate it at 37°C for 30 min. Mix by inversion every 5 min to make sure that the collagenase solution accesses the entire tissue and collect the solution and pass it through a strainer (40 μm) with the aid of a plunger, and resuspend the cells in 10 ml PBS and then centrifuge it at 800×g for 10 min. 3) Resuspend the pellet in 5 ml Ammonium-Chloride-Potassium lysing buffer. After 10 min at room temperature, add 10 ml of PBS to stop the reaction then centrifuge it at 800×g for 10 min. 4) Resuspend the pellet in RPMI-1640 medium and incubate the cells at 37°C for 2 h to let macrophages adhere to the dish. Shake the dish gently and discard the non-adherent cells. The adherent cells were rinsed three times with PBS. 5)

After trypsin digestion, the cells were harvested and resuspended in PBS. $CD14^+$ cells were isolated by CD14 MicroBeads (Miltenyi Biotec, Germany). The resulting cells were sorted further by FACS with specific monoclonal antibodies against CD14 (eBioscience, USA) and F4/80 (eBioscience, USA).

1.2. Expression and purification of rTcpC

The recombinant expression plasmid was transformed into *E. coli* BL21DE3 (Novagen, USA) for expression of rTcpC. *E. coli* strain $BL21DE3^{pET42a\text{-}\mathit{tcpc}}$ was cultured in LB liquid medium (Oxoid, UK) containing 50 μg/ml kanamycin (Sigma, USA) at 37°C in a shaker at 200 rpm. Expression of rTcpC was induced by 0.1 mM isopropyl-b-D-thiogalactoside (IPTG, Sigma). rTcpC was purified using a Ni-NTA affinity chromatographic column under NGC Chromatography System (Bio-Rad, USA). After concentrated by ultrafiltration, the purity of rTcpC was examined by SDS-PAGE plus an Agarose Image Analyzer (Bio-Rad). The possible contaminated *E. coli* LPS in the rTcpC was removed by Detoxi-gel column chromatography as described in our previous report [1]. Protein concentration of rTcpC was measured using a BCA Protein Assay Kit (Beyotime, China).

1.3. Removal of lipopolysaccharide in rTcpC

The possible contaminated *E. coli* LPS in rTcpC extract was removed with a Detoxi-gel endotoxin removing column chromatography (Thermo, USA) using pyrogen-free water for elution and then detected by using a Limulus Amebocyte lysate test kit (Lonza, Switzerland) as previously described [1].

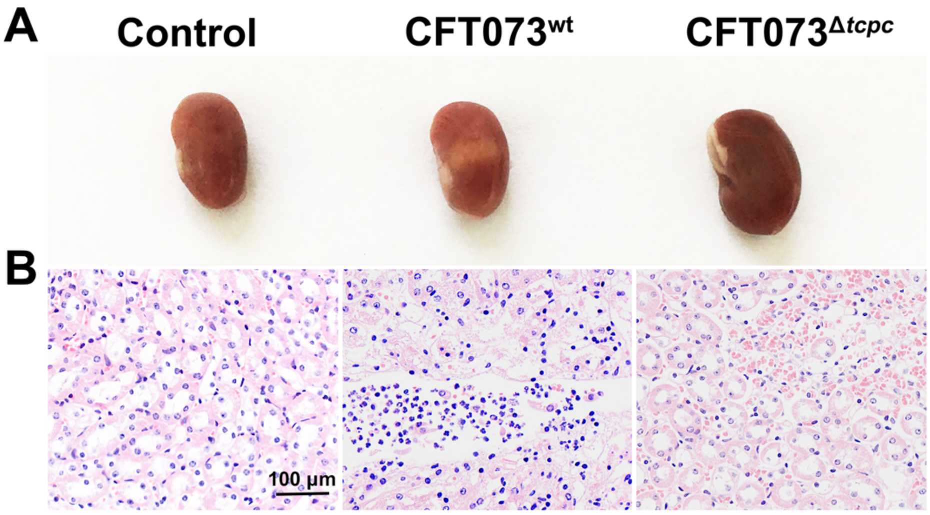

Figure S1. Abscesses and neutrophil infiltration could be observed in kidneys from CFT073^{wt}-induced mouse PN model.

(A) Gross observation of kidneys from CFT073^{wt}- or CFT073$^{\Delta tcpc}$-induced pyelonephritis mouse models. **(B)** Histological examination of kidneys from CFT073^{wt}- or CFT073$^{\Delta tcpc}$-induced pyelonephritis mouse models. Scale bar=100 μm.

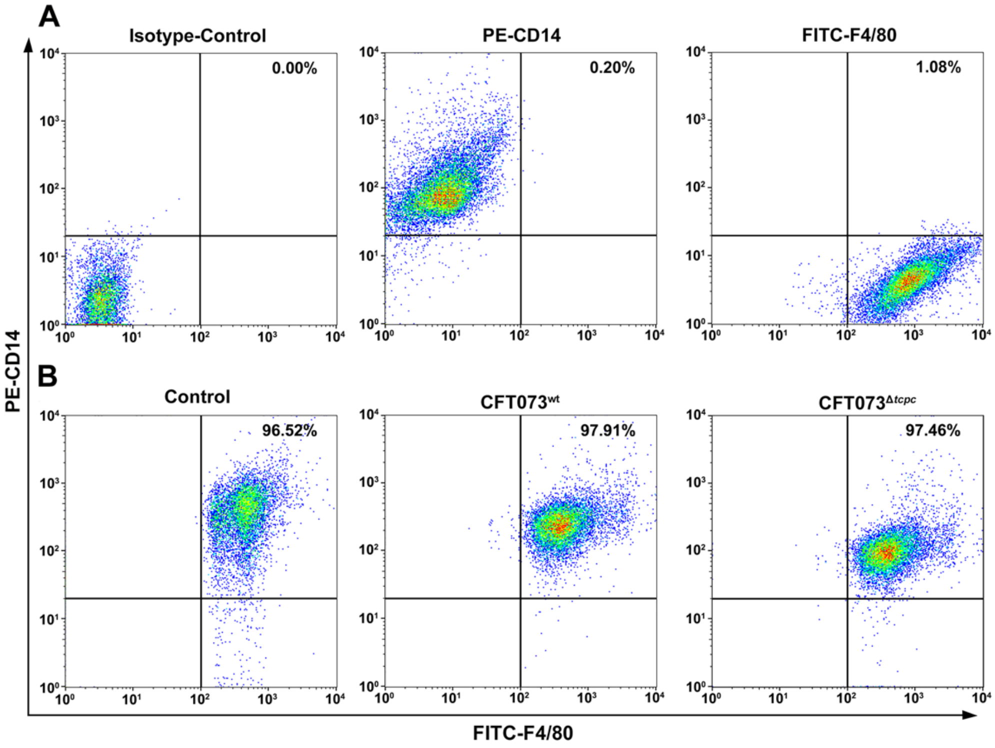

Figure S2. Purity identification of murine kidney macrophages.

(A) FACS profiles of isotype control (left), PE-CD14 monoclonal antibody (middle) and FITC-F4/80 monoclonal antibody (right). **(B)** FACS profiles of FITC-F4/80 and PE-CD14 monoclonal antibody double staining in K-macrophages isolated from normal control mice (left), CFT073^{wt}- (middle) and CFT073$^{\Delta tcpc}$-induced PN mouse models.

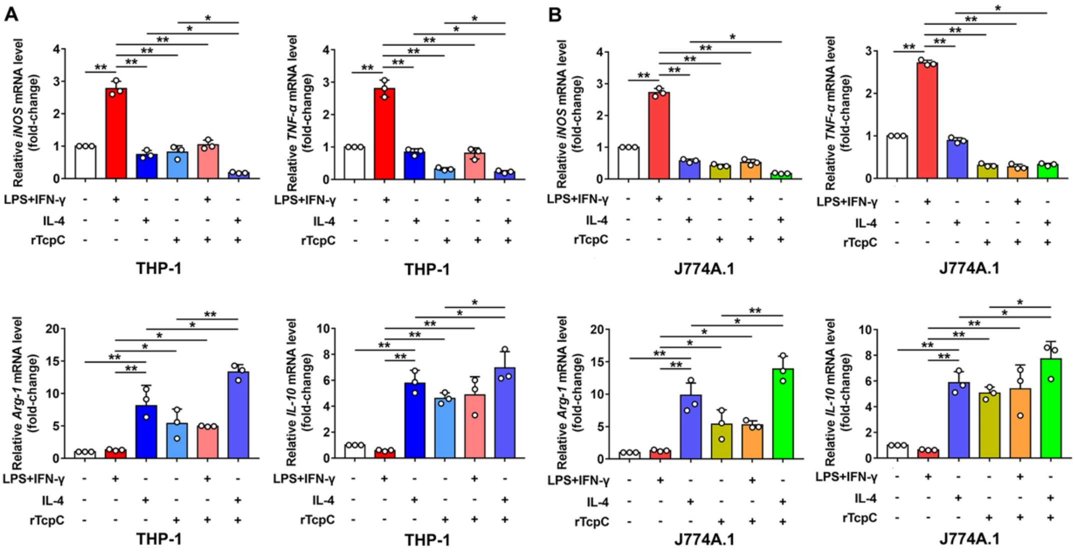

Figure S3. rTcpC inhibits the transcription of iNOS and TNF-α but promots the mRNA levels of IL-10 and Arg-1 in macrophages.

(A) mRNA levels of *iNOS*, *TNF-α, Arg-1*, *IL-10* in THP-1 were detected by qRT-PCR. **(B)** mRNA levels of *iNOS*, *TNF-α, Arg-1* and *IL-10* in J774A.1 were detected by qRT-PCR. Mean ± SD of three independent experiments were shown. *: p<0.05; **: p<0.01.

Table S1. Information of primers used in this study.

Primer	Sequence (5' to 3')	Purpose
m-iNOS	F: TTCTGTGCTGTCCCAGTGAG	Detection of mouse *iNOS* mRNA
	R: TGAAGAAAACCCCTTGTGCT	
h-iNOS	F: TCTTGGTCAAAGCTGTGCTC	Detection of human *iNOS* mRNA
	R: CATTGCCAAACGTACTGGTC	
m-Arg-1	F: TTTTTCCAGCAGACCAGCTT	Detection of mouse *Arg-1* mRNA
	R: AGAGATTATCGGAGCGCCTT	
h-Arg-1	F: TCCAAGCCAAAGTCCTTAGAGATTAT	Detection of human *Arg-1* mRNA
	R: CGTCATACTCTGTTTCTTTAAGTTTTC	
IL-10	F: ATTTGAATTCCCTGGGTGAGAAG	Detection of *IL-10* mRNA
	R: CACAGGGGAGAAATCGATGACA	
m-TNF-α	F: ATGAGCACAGAAAGCA TGATCCGC	Detection of mouse *TNF*-α mRNA
	R: AAAGTAGACCT GCCCGGACTC	
h-TNF-α	F: CCTGTAGCCCACGTCGTAG	Detection of human *TNF-α* mRNA
	R: GGGAGTAGACAAGGTACAACCC	
m-Actin	F: CTCCATCCTGGCGCTGT	Mouse inner reference
	R: GCTGTCACCTTCACCGTT	
h-Actin	F: ATGGATGACGATATCGCTG	Human inner reference
	R: AACACCCATTCCCTTCACAG	

F: forward primer, R: reverse primer.

Reference

1. Fang, J.Q., Q. Ou, J. Pan, J. Fang, D.Y. Zhang, M.Q. Qiu, Y.Q. Li, X.H. Wang, X.Y. Yang, Z. Chi, et al. TcpC inhibits toll-like receptor signaling pathway by serving as an E3 ubiquitin ligase that promotes degradation of myeloid differentiation factor 88. *PLoS. Pathog.* **2021**, *17*, e1009481.
